# Multicentric lymphoma in a donkey with intestinal and bone marrow involvement

**DOI:** 10.1186/s12917-024-03903-5

**Published:** 2024-02-15

**Authors:** Georgios Paraschou, Cynthia Xue, Rebecca Egan, Pompei Bolfa

**Affiliations:** 1 Department of Biomedical Sciences, Ross University School of Veterinary Medicine, Basseterre, Saint Kitts And Nevis; 2 Department of Clinical Sciences, Ross University School of Veterinary Medicine, Basseterre, Saint Kitts And Nevis; 3 Animal Health Laboratory, University of Guelph, Guelph, ON Canada

**Keywords:** Bone marrow, Donkey, Equine, Intestine, Lymphoma, Multicentric

## Abstract

**Background:**

Lymphoma is a common neoplasm in horses but is reported much less commonly in donkeys. In this case report, we describe the macroscopic, microscopic and immunohistochemical features of a multicentric lymphoma with intestinal and bone marrow involvement.

**Case presentation:**

A geriatric female donkey with history of chronic lameness was found dead. Post-mortem examination revealed advanced emaciation, periodontal disease, left front foot laminitis and multiple, soft, white to yellow tan intestinal transmural masses, up to 12 cm in diameter. Cytology suggested a round cell intestinal neoplasm. The femur of the left hint limb was double the size of the normal contralateral, with suspected neoplastic infiltration and replacement of bone marrow and bone. Histologically we diagnosed a lymphoma in the intestine and left femur. Immunohistochemically, the neoplastic cells showed CD3 immunolabelling, supporting a diagnosis of a multicentric T-cell lymphoma.

**Conclusions:**

To the authors’ knowledge, this is the first time multicentric lymphoma is diagnosed in donkeys. Further studies of the genetic background, clinical, laboratory, histopathologic, and immunohistochemical, as well as the pathogenesis of lymphoma, is needed to better understand the uniquely low frequency of this neoplasm in donkeys.

## Background

Equine lymphoma and squamous cell carcinoma are the most common malignant neoplasms in horses [1–5], with lymphoma being the most common malignant neoplasm found in the alimentary tract of the horse [1, 5]. Lymphoma in horses is classified by anatomic distribution into alimentary, cutaneous, multicentric, or mediastinal forms, with multicentric being the most common followed by cutaneous and alimentary [2]. Most common histologic types of lymphomas in horses are T-cell rich large B-cell lymphoma (TCRLBCL), diffuse large B-cell lymphoma and peripheral T-cell lymphoma [2]. Alimentary lymphoma of horses is predominantly of T-cell origin, with enteropathy associated T-cell lymphoma (EATL) being the most diagnosed histologic type [1].

Unlike horses, it appears that lymphoma is not a commonly reported neoplasm in donkeys, with only 3 confirmed and 1 presumed/unconfirmed published cases, with the confirmed cases being: an unclassified (no immunohistochemistry performed) alimentary lymphoma, a cutaneous epitheliotropic T-cell lymphoma, and a pulmonary angiocentric lymphoma [4, 6, 7]. In two large retrospective reviews, one of neoplasia in 125 donkeys in the United States of America and Canada and one of autopsy cases from 1,404 geriatric donkeys in the United Kingdom, only one presumed and unconfirmed case of lymphoma in peripheral lymph node without further diagnostic investigation is mentioned [8, 9].

## Case presentation

A geriatric jenny based on dentition was submitted to the pathology department at Ross University School of Veterinary Sciences in Saint Kitts after being found dead in the pen by the caretaker. The donkey was found deceased approximately 2 days prior to autopsy, during which time the body was refrigerated. Some of the cytological and histological features were affected by this delay between the death and the post-mortem examination, but histology and immunohistochemistry (IHC) remained feasible. The jenny was initially acquired as an adult from a feral herd of donkeys on the neighbouring island of Nevis approximately 3 years prior for integration into the research herd. At the time of acquisition, complete blood count and serum biochemistry analysis did not reveal abnormalities consistent with systemic illness. This jenny was anecdotally noted to have a history of chronic intermittent lameness, but no further characterization or workup of the lameness appeared within the medical record of the patient.

**Fig. 1:**
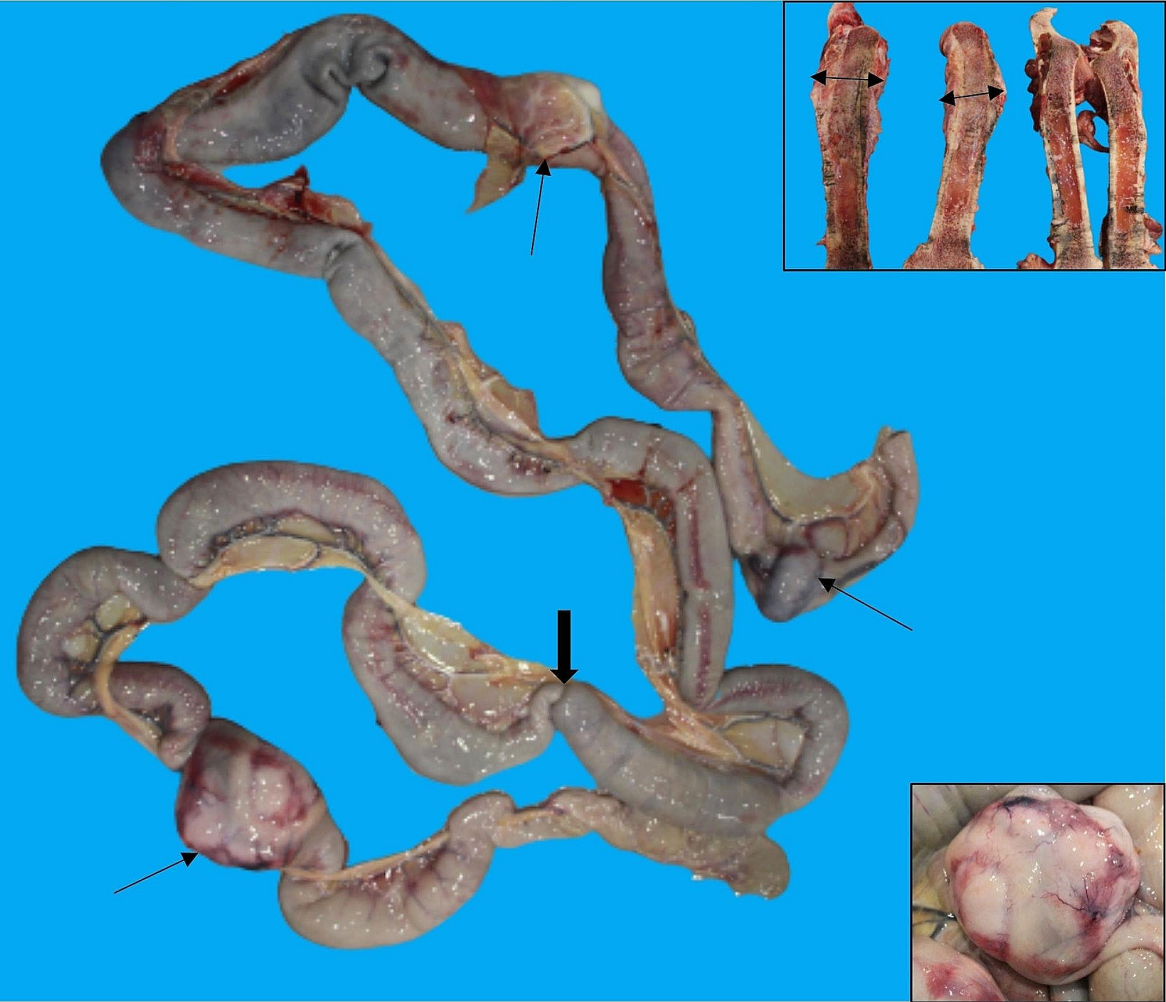
Donkey, jejunum. Multiple nodular yellow-tan, transmural masses (thin arrows) of intestinal lymphoma. Intestinal intussusception (thick arrow), in proximity of a lymphoma nodule. Upper right inset: left femur (first 2 bone sections on the left of the image), proximal metaphysis and diaphysis expanded and effaced by a yellow to white mass (double headed arrows) compared to right femur which is shown as control. Lower right inset: higher magnification of an intestinal lymphoma nodule

At autopsy, the donkey was found to be emaciated with serous atrophy of pericardial and long bone marrow adipose tissue, generalized muscle atrophy, and hepatic atrophy, in addition to having severe chronic periodontal disease with periodontal pockets, cheek teeth diastemata, alveolar bone retraction, an overgrown and deformed left front hoof, and catarrhal rhinitis and bronchitis with one intraluminal adult nematode consistent with *Dictyocaulus arnfieldi*. One of the most significant gross findings was seen in the small intestine at the level of the jejunum, where there were multiple, up to 12 cm in diameter, nodular, irregular, white to yellow-tan, soft, friable masses infiltrating the intestinal wall and often causing intestinal stenosis (Fig. 1). The intestinal mucosa overlying some of the masses was ulcerated, and a secondary intussusception was identified adjacent to one mass (Fig. 1, inset). An impression smear of one of the masses was obtained at the time of autopsy and stained with Diff quick, and cytologic examination revealed a heavily cellular sample consisting mainly of poorly preserved monomorphic round cells. In the left femur, the proximal metaphysis and adjacent diaphyseal area were markedly enlarged, up to approximately twice the diameter of the right femur, and the bone marrow cavity was infiltrated by a firm mass of white to yellow tissue that was obscuring and disrupting the metaphyseal trabecular bone and the bone marrow, accompanied by extensive periosteal remodelling. Left femoral lesions were initially interpreted as metastatic neoplasia, inflammation or a combination.

**Fig. 2:**
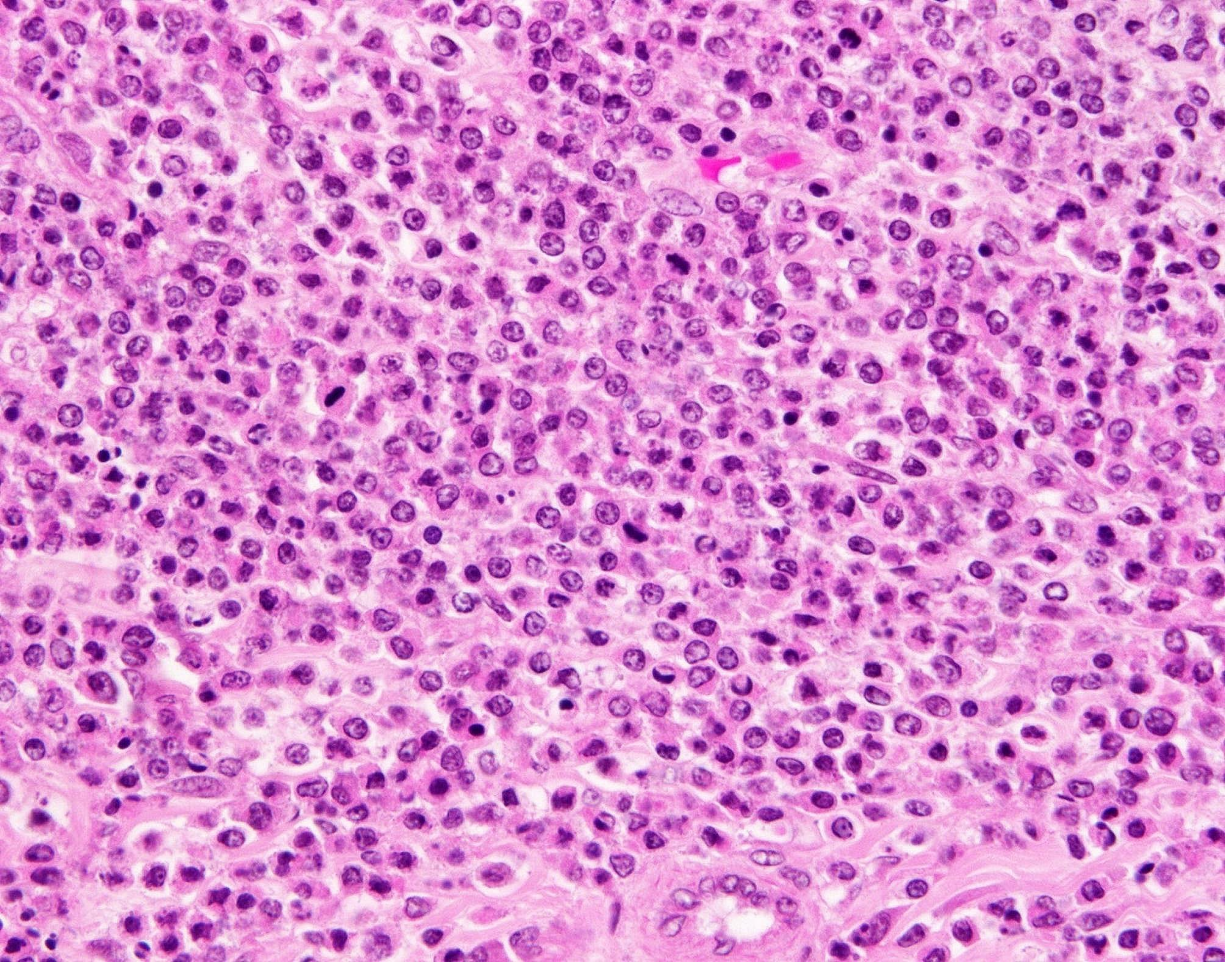
Donkey, jejunum, T-cell lymphoma. The intestinal wall is expanded and effaced by sheets of round cells exhibiting mild anisocytosis and anisokaryosis. HE

**Fig. 3:**
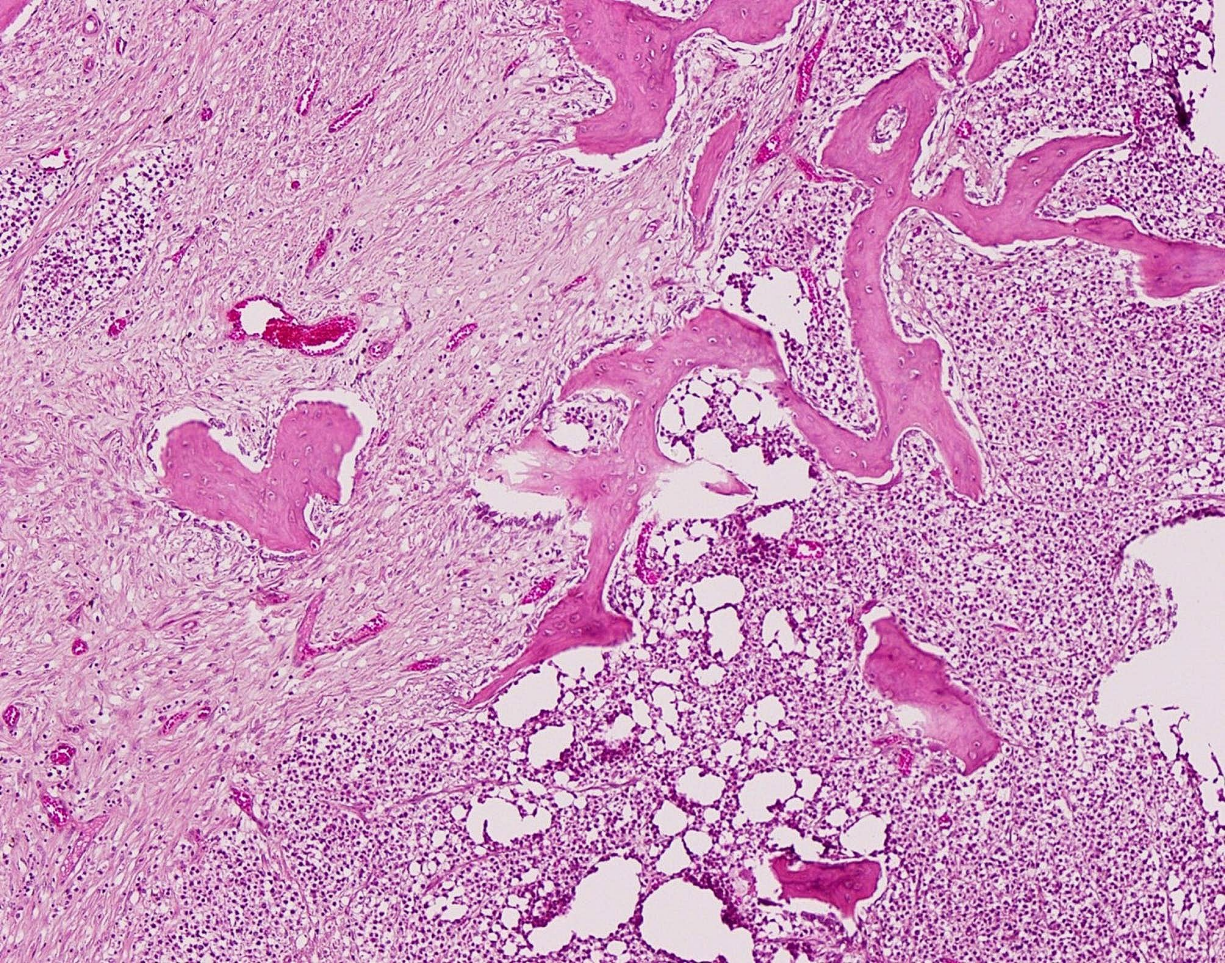
Donkey, proximal femur, diaphysis, T-cell lymphoma. The bone marrow is infiltrated, expanded and replaced by neoplastic round cells (right side) and fibrous connective tissue (left side). Trabecular bone is subject to extensive remodelling. HE

Representative tissue samples were fixed in 10% neutral-buffered formalin, processed routinely in paraffin, sectioned, and stained with haematoxylin and eosin (HE) for microscopic examination. Histologic examination of the jejunum revealed transmural infiltration and effacement of all layers of the intestine by monomorphic population of neoplastic round cells (compatible with a lymphoma) with distinct cytoplasmic borders and supported by a fine, pre-existing fibrovascular stroma (Fig. 2). Neoplastic cells exhibited a high nuclear to cytoplasmic ratio and contained a single, round hyperchromatic nucleus with one to two nucleoli. There was mild anisocytosis and anisokaryosis with up to five mitotic figures per 2.37mm^2^. Neoplastic cell aggregates compatible with tumour emboli were identified within veins of the tunica muscularis, suggesting lymphovascular invasion. Expanding and replacing the bone marrow and cortical bone, and extending into the periosteum of the proximal left femur, there was a round cell neoplasm with similar features to the one present in the small intestine (Fig. 3). In addition, multifocal replacement of the bone marrow with fibrous connective tissue and periosteal bone remodelling with increased osteoclastic activity were apparent in the area with the neoplasm (Fig. 3).

**Fig. 4:**
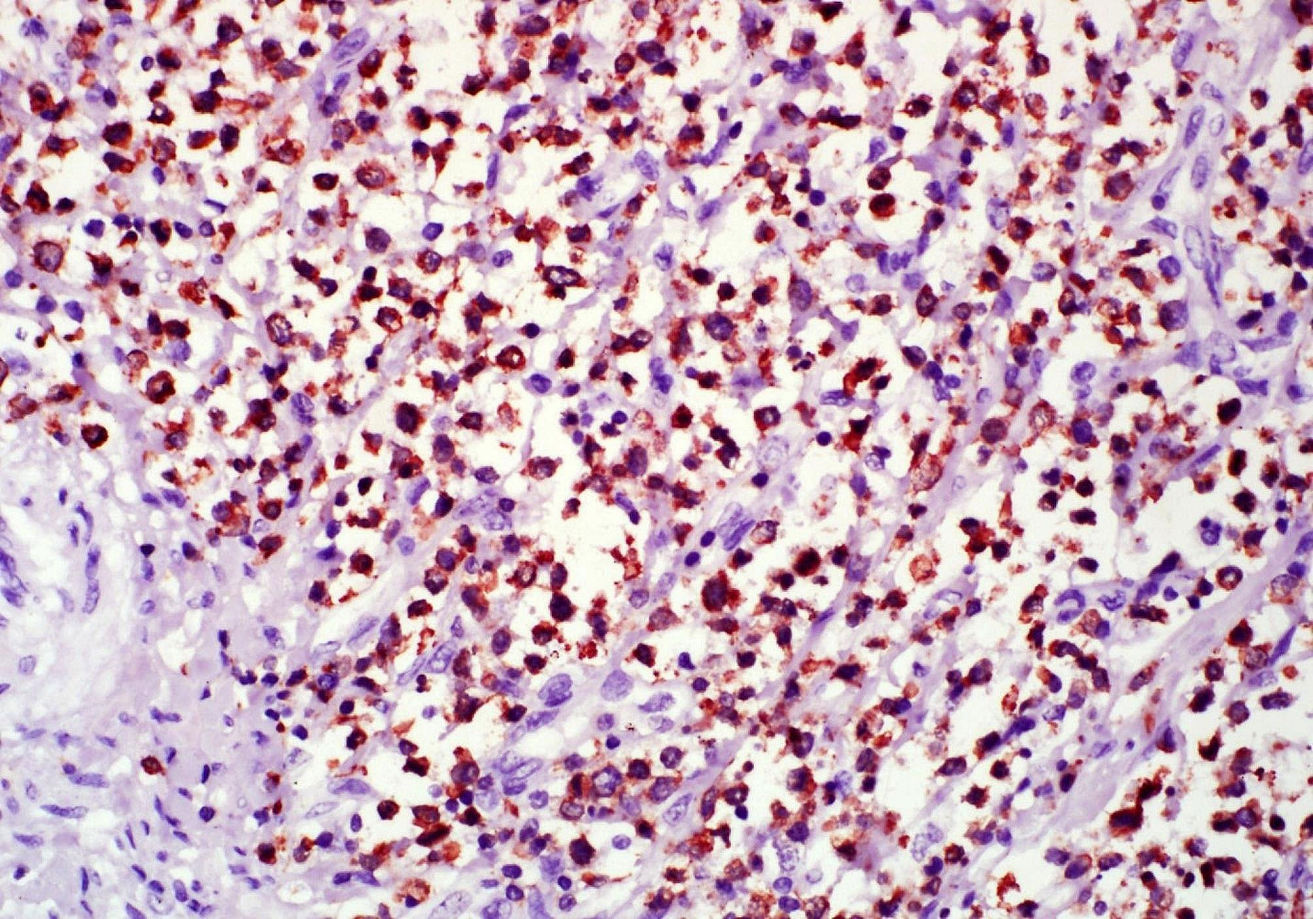
Donkey, jejunum, IHC for CD3. Diffuse and strong membranous and cytoplasmic immunolabelling of neoplastic round cells

Immunohistochemistry was pursued to further characterize the neoplastic round cell population. Immunohistochemical staining for CD3 and CD79a was performed using rabbit polyclonal anti-human CD3 and mouse monoclonal antihuman CD79a (Dako, Denmark), and species cross-reactivity of both antibodies has been demonstrated in various species, including horses. In the intestinal mass, the majority of neoplastic cells exhibited moderate to intense cytoplasmic and lesser membranous immunoreactivity for CD3 (Fig. 4). Randomly scattered throughout the mass, the CD3 immunolabelled cells were intermingled with individual round cells exhibiting immunoreactivity for CD79a. Similarly, the majority of round cells comprising the infiltrative population in the bone marrow exhibited membranous and/or cytoplasmic immunoreactivity for CD3, and these were accompanied by scattered individual cells exhibiting immunoreactivity for CD79a. Combined interpretation of histologic and immunohistochemical findings supports a diagnosis of a multicentric lymphoma with features compatible with a T-cell lymphoma..

## Discussion and conclusions

Although lymphoma is the most common malignant neoplasm in horses, there is paucity of literature on this neoplasm in donkeys [1–4, 6–9]. The most common neoplasm shared with horses and donkeys is sarcoids, whilst other neoplasms that appear more frequently in horses seemingly affect donkeys less commonly [8]. The most common anatomic and histologic types of lymphoma in horses are multicentric and T-cell-rich large B-cell lymphoma (TCRLBCL), respectively [2]. The most common histologic type of alimentary lymphoma in horses is EATL similar to those seen in dogs, cats and humans [10–13]. Currently, only three case reports of lymphoma in donkeys have been described in the literature: an unclassified (no Immunohistochemistry performed) alimentary lymphoma, a cutaneous epitheliotropic T-cell lymphoma, and a pulmonary angiocentric lymphoma [4, 6, 7]. With such a low number of cases, no substantial conclusions can be made in terms of the type of lymphoma most common in this species. In this case report, a multicentric lymphoma with involvement of the small intestine and the bone marrow in a geriatric jenny is described. The scarcity of lymphomas in donkeys is poorly understood. It may be that the suspected retroviral aetiology of lymphoma in horses [14, 15] is not common in donkeys. In horses, much effort has been made to describe clinical, laboratory, histopathologic and immunohistochemical features, efficacy of treatment and survival rates of lymphoma [1, 16, 17]. However, such characterizations are not as thorough in the literature regarding donkey lymphomas likely due to scarcity of cases as well as economic limitations precluding further evaluation of the neoplasm. Interestingly, although neoplasia is the most common cause of death in dogs and the second most common cause in humans (USA), it is not one of the top 5 common causes of death in the horse [18]. Interspecies comparison between donkeys and horses on common causes of mortality cannot be performed at this time as there is a lack of similar prevalence of surveys in donkeys.

The major contributors to the poor body condition of the donkey in the current of case included multicentric lymphoma with intestinal and bone marrow involvement, a hoof deformity, periodontal pockets and cheek teeth diastemata with associated periodontal disease. Cachexia, as seen in this donkey, is the most common paraneoplastic syndrome of malignancy, suggested by the weight loss, hepatic and muscle atrophy and serous atrophy of fat. While the malignancy may be a large element of the donkey’s poor body condition, someone cannot overlook the contribution of chronic periodontal and hoof disease to the donkey’s cachexia. Although a more robust clinical monitoring program has been implemented recently at Ross University School of Veterinary Medicine, little is known about this donkey’s history including the duration of weight loss and presence of dental and hoof disease.

We retrieved no cases of multicentric lymphoma in donkeys in a search of multiple databases, suggesting that no descriptions of this condition have been reported in donkeys to date. Further studies of the genetic background, clinical, laboratory, histopathologic, and immunohistochemical, as well as the pathogenesis of lymphoma, is needed to better understand the uniquely low frequency of an otherwise aggressive and common neoplasm in horses, in donkeys.

## Data Availability

Data sharing is not applicable to this article as no datasets were generated or analysed during the current study.

## Abbreviations

TCRLBCL: T-cell rich large B-cell lymphoma
EATL: Enteropathy associated T-cell lymphoma
IHC: Immunohistochemistry
HE: Haematoxylin and eosin
CD3: Cluster of differentiation 3
CD79a: Cluster of differentiation 79a
